# PHF20 Promotes Glioblastoma Cell Malignancies Through a *WISP1*/*BGN*-Dependent Pathway

**DOI:** 10.3389/fonc.2020.573318

**Published:** 2020-10-06

**Authors:** Qianquan Ma, Wenyong Long, Changsheng Xing, Chongming Jiang, Jun Su, Helen Y. Wang, Qing Liu, Rongfu Wang

**Affiliations:** ^1^Department of Neurosurgery in Xiangya Hospital, Central South University, Changsha, China; ^2^Department of Neurosurgery in the Third Hospital of Peking University, Peking University, Beijing, China; ^3^Center for Inflammation and Epigenetics, Houston Methodist Research Institute, Houston, TX, United States; ^4^Department of Medicine, Keck School of Medicine, University of Southern California, Los Angeles, CA, United States; ^5^Department of Pediatrics, Children’s Hospital of Los Angeles, Keck School of Medicine, University of Southern California, Los Angeles, CA, United States; ^6^Norris Comprehensive Cancer Center, Keck School of Medicine, University of Southern California, Los Angeles, CA, United States

**Keywords:** PHF20, glioblastoma, WISP1, BGN, cancer stem cell-like traits, epigenetic regulation

## Abstract

Glioblastoma (GBM) stem cells are resistant to cancer therapy, and therefore responsible for tumor progression and recurrence after conventional therapy. However, the molecular mechanisms driving the maintenance of stemness and dedifferentiation are poorly understood. In this study, we identified plant homeodomain finger-containing protein 20 (PHF20) as a crucial epigenetic regulator for sustaining the stem cell-like phenotype of GBM. It is highly expressed in GBM and tightly associated with high levels of aggressiveness of tumors and potential poor prognosis in GBM patients. Knockout of PHF20 inhibits GBM cell proliferation, as well as its invasiveness and stem cell-like traits. Mechanistically, PHF20 interacts with WDR5 and binds to the promoter regions of WISP1 for its expression. Subsequently, WISP1 and BGN act in concert to regulate the degradation of β-Catenin. Our findings have identified PHF20 as a key driver of GBM malignant behaviors, and provided a potential target for developing prognosis and therapy.

## Introduction

Glioblastoma (GBM) is the most malignant primary tumor of the central nervous system, accounting for about 70% of intracranial primary gliomas in adults. Despite modern neurosurgery and intensive conventional therapy, GBM patients have limited treatment options with high rates of relapse. The average survival time of GBM patients is less than 16 months, and the overall 5-year survival rate is less than 10% (1). Thus, it is of great importance to identify key regulators that control GBM risk stratification, to aid in the development of more effective therapeutic drugs.

Cancer stem cells (CSCs) have been widely recognized as a key feature of GBM (2, 3). Recent studies show that CSCs extensively affect tumor growth, drug resistance, and recurrence and are closely related to the prognosis of patients (4–6). As such, elucidating the mechanism of GBM stem cell (GSC) proliferation and maintenance is critical to improve our understanding of the development of GBM. Previous studies have shown that the exogenous overexpression of four essential factors (POU3F2, SOX2, SALL2, and OLIG2) is sufficient to fully reprogram differentiated GBM cells and induce poorly differentiated GBM stem-like cells (7). Consistently, poorly differentiated gliomas have significantly worse clinical prognosis than well-differentiated gliomas. Therefore, targeting GSCs is a promising approach for the development of novel GBM therapies.

Plant homeodomain-finger containing protein 20 (PHF20) has been previously identified as a novel antigen in glioma patients and named as glioma-expressed antigen 2 (GLEA2) (8, 9). PHF20 functions as a key epigenetic regulator of stem cell self-renewal and cellular reprogramming (10), and is abundantly expressed in several cancers (11–14). Based on the similarities between somatic cellular reprogramming and cancer stem cells reprogramming, PHF20 plays an important role in carcinogenesis by dramatically enhancing the self-renewal and tumor-initiating capabilities of lung cancer cells, as well as controlling the stem cell-like phenotype of neuroblastoma cells (15, 16). Intriguingly, it had also been reported that the expression level of PHF20 was significantly associated with the pathological grade of glioma (17). However, the role of PHF20 in GBM remains largely unknown. In this study, we report that PHF20 is highly expressed in GBM and inversely associated with the potential prognosis of GBM patients. Ablation of PHF20 inhibited the proliferation and malignancy, while ectopic expression of PHF20 enhanced the expression of WISP1 and BGN, resulting in the formation of a complex between WISP1 and BGN that regulated the degradation of β-Catenin, suggesting that PHF20 is a pivotal factor of GBM development. Thus, our findings have discovered PHF20 as a therapeutic target for GBM therapy.

## Materials and Methods

### Cell Lines and Cell Culture

All of the BT cell lines (BT115, BT135, BT136, BT139, BT141, BT145, BT147, BT149, BT150, and BT156) were given as gifts from Neurosurgery Department, Houston Methodist Hospital. All these cell lines were primarily cultured from patients with GBM, which were histopathologically diagnosed at the Houston Methodist Hospital. Among these BT GBM cell lines, the BT115 cell line was considered to be the most malignant one based on the clinical information, the patient survived only several months after surgery, because of the recurrent of tumor. During the culture of these cell lines, we also found the BT115 cells grew fast and were highly aggressive, while the other BT cells grew extremely slow even hard to passage. From this perspective, we selected BT115 cell line for further experiment. The human U87 cell line as a standard GBM cell line, which was obtained from American Type Culture Collection (ATCC) and was certified by STR analysis. BT115 and U87 cell lines were grown in Dulbecco’s modified Eagle’s medium (DMEM) containing 10% fetal bovine serum (FBS) at 37°C in a humidified 5% CO_2_ atmosphere. The negative control cells were normal glial cell line HEB cells, while induced pluripotent stem cells (iPS cells) were used as positive control cells.

### Generation of *PHF20* KO Cell Lines

BT115 and U87 cells were stably transfected with PHF20 sgRNA (pLentiCRISPR V2). PHF20 knockout (KO) cells were identified by limiting dilution cloning. Briefly, the cells were plated at a density of 3 × 10^5^ cells per 6-well plate. Glioma cells were, respectively, transfected with control sgRNA or PHF20 sgRNA expression lentivirus. Two days after transfection, 2 μg/ml puromycin was added into the culture medium for 3 days. Then, the cells were transferred to a new medium containing 2 μg/ml puromycin at a density of 0.3 cells per well in 96-well plates. After three weeks, 10–30 single clones per sgRNA were picked and expanded. The efficiency of PHF20 KO of the resulting single clones was examined by western blot analysis.

### WISP1, BGN, and WDR5 shRNA Gene Silencing

WNT1 inducible signaling pathway protein 1, BGN, WDR5, and non-specific control lentiviral shRNAs were obtained from the GIPZ shRNA library. BT115 and U87 cells were transfected with lentiviruses harboring different shRNAs. Prior to use, shRNA-positive cells were validated green fluorescence microscope and selected for by culturing in medium containing 2 μg/ml puromycin for 1 week.

### Gene Rescue Experiment

For PHF20 gain-of-function experiments, the human PHF20 (NM_016436.4) cDNA sequence was cloned into a pLV-lentiviral vector. The Teton lentiviral vector (pTet-DEST-Flag-targetgene-puro + pLenti-rtTA-ZEO) was co-transfected with the VSVG and PAX2 lentiviral packaging vectors into 293T cells. The supernatants with lentiviruses were collected on day 3 and concentrated by ultra-centrifugation. The concentrated lentiviruses were then re-suspended in 1 ml of PBS. *PHF20* KO cells were infected with Teton lentiviruses harboring PHF20 and generated ectopically re-expressed PHF20 in *PHF20* KO cells. For *WISP1* and *BGN* rescue, *WISP1/BGN* knock-down (KD) cells were infected with a Teton plasmid harboring *WISP1* or *BGN* or *WISP1* and *BGN*. As a result, we enforced ectopically expressed *WISP1* and *BGN* alone or together in WISP1/BGN KD cells. The expression of each gene was ectopically induced by doxycycline treatment (0.1 μg/ml). Cells transfected with Teton plasmid without doxycycline treatment were used as control.

### Cell Viability Assay

An MTT assay was used to check the tumor cell viability. Cells were cultured in 96-well plates at a density of 1 × 10^3^ cells/well before incubating at 37°C in a humidified 5% CO_2_ atmosphere. The culture medium was removed at six time points (0, 24, 48, 72, 96, and 120 h). Each well of cell lysis was added 20 μl of MTT solution and incubated for 4 h. After 4 h, the incubation buffer was discarded, and the blue MTT-formazan product was extracted from each well by adding 100 μl DMSO. The 96-well plates were covered with aluminum foil and shaken for 15 min. The absorbance of formazan solution was then read spectrophotometrically at 540 nm in 1 h.

### Transwell Invasion and Migration Assay

The invasiveness of cells was examined by their activities to pass through Corning Matrigel. In the beginning, the upper surface of the polycarbonic membranes (8.0 μm pore size) of the transwell chambers were coated with Matrigel (1:4 diluted with RPMI 1640). Cells (3 × 10^4^) in 100 μl of RPMI 1640 with 2% FBS were seeded into the upper chambers. The lower compartments of the chambers were added with 500 μl of RPMI 1640 containing 10% FBS. 48 h later, the migrated cells from the Matrigel to the lower surface of the chambers were fixed in 70% ethanol and stained with 0.2% crystal violet. The number of cells were counted under microscope (100× magnification). Cell invasiveness was assessed by averaging the number of cells counted in four randomly selected visual fields per chamber.

### Neurosphere Assay

Cells were cultured (3000 cells/well) in complete neural stem cell (NSC) basal medium [9:1 mixture of NSC basal medium and NSC proliferation supplement containing 20 ng/ml EGF, 10 ng/ml basic fibroblast growth factor (bFGF), and 1 μl/ml of 0.2% heparin] in 24-well ultralow-attachment plate (Corning Life Sciences, Union City, CA, United States). For this experiment, 1 ml of the medium was used in each of the 24 wells as the stem cell medium (SCM). After 72 h of incubation, the sphere number and size were counted for analysis. The neurospheres are considered as the clonal cell clusters of neural stem cells, thus neurosphere assay has been widely used in neurobiological research.

### Real-time PCR, RNA-Sequencing and Bioinformatics Analysis

Complementary DNA was obtained from the tumor cells total RNA using SuperScript II Reverse Transcriptase (Invitrogen) with oligo (dT) primers. The primer sequences for the target genes were designed using Primer BLAST software and were presented in Supplementary Table 2. Quantitative PCR was then performed using QuantStudio 6 Flex Real-Time PCR System and Power SYBR Master Mix. The relative mRNA expression level was analyzed using the 2^–ΔΔCt^ method; *GAPDH* was used as an endogenous control to normalize Ct values.

For RNA sequencing analysis, two independent groups of BT115 *PHF20* KO (PHF20 sgRNA + PHF20 Teton, without doxycycline), BT115 *PHF20* Teton (PHF20 sgRNA + PHF20 Teton, with doxycycline), and BT115 control (control sgRNA) cells were firstly validated by western blot analysis. Then, cells lysed by Trizol and RNA were generated. Each sample was diluted in 200 μl RNase-free ddH_2_O. The concentration of each sample was greater than 300 ng/μl. The BT115 samples were sent to Novogene Company for RNA-sequencing. Poly(A)-selected RNA libraries were prepared using the Illumina TruSeq library construction, and resulting libraries were sequenced on an Illumina HiSeq machine using 150-bp paired-end reads. Reads were aligned to the reference human genome (GRCh37) using TopHat version 2.0.12 (18). Next, the realigned.bam files were sorted by name using SAMtools version 1.9 (19). HTSeq was used to count the number of reads mapping to each gene with version 0.6.1p1 (20).

The RNA-sequencing data was analyzed by following rules:

1. Genes which had following characters (log2 fold change >5 or <−5 in “*PHF20* KO group vs *PHF20* control group”; log2 fold change in *PHF20* Teton group> or <control group) were directly selected for functional validation.
2. Genes which had fold change > |2| with linear expression tendency were selected for further KEGG pathway analysis. Data normalization for differential expression analysis was carried out with the DEGSeq version 1.12.0, |log2foldchang| > = 2 and padj < = 0.05 (21). The heatmap was created using the pheatmap R package, version 1.0.12^1^. Pathway enrichment analysis was carried out by using DAVID functional annotation tool to compare the differentially expressed genes to KEGG gene sets, *P* < = 0.05 was used as cutoff (22, 23).

### Western Blotting, Immunoprecipitation (IP), and Mass Spectrometry

Cells were lysed in either low salt lysis buffer or RIPA buffer containing protease inhibitors. Equal amounts of protein samples were separated electrophoretically and then transferred onto PVDF membranes (Bio-Red, Hercules, CA, United States). The membranes were blocked for 1h in Tris-buffered saline Tween-20 (TBST) with 5% non-fat milk. Thereafter, western blot analysis was performed using primary antibodies against PHF20 (1:1000, Cell Signaling, Danvers, MA, United States), WDR5 (1:1000, Cell Signaling, Danvers, MA, United States), WISP1 (1:1000, Sigma), BGN (1:2000, Sigma), β-Catenin (1:1000; Cell Signaling, Danvers, MA, United States), and β-actin (1:2000; Cell Signaling, Danvers, MA, United States) in a blocking buffer containing 5% non-fat milk and 0.1% Tween-20 in TBS. The blots were then developed using Lumiglo substrate (KP Laboratories, Gaithersburg, MD, United States) on BioMax LS film (Eastman Kodak, Rochester, NY, United States).

For IP, samples were centrifuged at 14,000 × *g* for 15 min at 4°C. The supernatant was added with either a 10 μl anti-Flag M2 affinity gel or primary antibody and rotated at 4°C overnight. After incubation with immobilized Protein A 16 (Repligen, Waltham, MA, United States), low salt lysis buffer was used to wash samples for five times. The proteins were then re-suspended in 2 × SDS sample loading buffer and subjected to SDS/PAGE. The resolved proteins were transferred to nitrocellulose (NC) membranes for immunoblotting.

### Human Datasets and Survival Analysis

Publicly available and freely accessible online cancer data include GEO (NCBI Gene Expression Omnibus) and TCGA (The Cancer Genome Atlas) were used in this study. In brief, the samples within a dataset were sorted according gene expression and subsequently divided into two groups (including genes like ITGB2, CADM1, LTBR, WISP1, BGN, and β-catenin) low and high based on the X-tile cutoff expression value. All cutoff expression levels and their resulting groups were analyzed for survival. The best *P*-value and the corresponding cutoff value were selected to generate Kaplan-Meier graphs.

### Subcutaneous Tumor Model

All animal experiments were approved by the Institutional Animal Care and Use Committee (Houston Methodist Research Institute).

For the *in vivo* tumor formation experiments, eight-week old NSG mice were divided into two groups of four mice and five mice (one group for *PHF20* KO cells and another group for control). As the limitation of our *in vivo* experiments, intracranial orthotopic model could not been conducted at this time. Each mouse was subcutaneously injected in the right flank with 3 × 10^5^ U87 cells (with PHF20) diluted in 500 μl of 50% PBS/50% Matrigel. Every two days, the size of tumors was checked by measuring their length and width. The tumors were harvested at 26 days. The tumor volume was calculated with the following formula: volume (mm^3^) = (length^∗^width^∗^width)/2. The brain and lung tissues were removed, fixed in formalin, and stored at 4°C.

### Immunohistochemistry (IHC)

Paraffin-embedded human glioma samples from Xiangya hospital were resected. The study cohort consisted of 23 cases, including 5 cases of normal brain tissues, and 3, 5, 4, and 6 cases of WHO grade I-IV glioma tissues. The tumor tissues were formalin-fixed, processed, and paraffin-embedded. Antigens were retrieved by autoclaving in 0.01 mol/l sodium citrate buffer (pH 6.0) at 121°C, 20 psi for 3−5 min. Endogenous peroxidase activity and non-specific binding sites were blocked using 3% hydrogen peroxide and 5% goat serum, respectively. The blocked sections were incubated overnight at 4°C with primary antibody followed by 30 min incubation with secondary antibody. The slides were stained with diaminobenzidine (DAB) for 2 min, counterstained with hematoxylin, and mounted with Immuno-mount (Thermo Fisher Scientific). The scoring criterion was taken as the average percentage of positively stained cells counted in ten randomly selected visual fields. IHC was performed with primary antibodies against PHF20 (1:100, Sigma-Aldrich), Ki-67 (1:500, Cell Signaling, Danvers, MA, United States), NESTIN (1:200, Cell Signaling, Danvers, MA, United States), WISP1 (1:50; Sigma, Danvers, MA, United States), BGN (1:200; Sigma, Danvers, MA, United States), and β-Catenin (1:1000; Cell Signaling, Danvers, MA, United States). The IHC staining and quantification were completed by two blind individuals.

### ChIP-PCR

The reader should refer to the Thermo Fisher ChIp Kit Manual Book.

### Statistical Analysis

GraphPad Prism version 5.0 was selected for all statistical analyses. Data are showed as the mean ± standard deviation (SD) of three independent experiments. The relationship between PHF20 expression in human glioma tissues and tumor grades was analyzed using Spearman’s rank correlation coefficient test. Two groups comparisons were performed using two-sided Student’s *t*-test. For experiments with three groups or more, the non-parametric Mann-Whitney *U* (MWU) test was used for comparisons between target groups and the results are obtained by means of the MWU test. For all tests, a *P*-value < 0.05 was considered statistically significant.

## Results

### PHF20 Is Highly Expressed in GBM, and Increases Cellular Viability, Proliferation and Invasiveness of GBM Cells Both *in vitro* and *in vivo*

**FIGURE 1:**
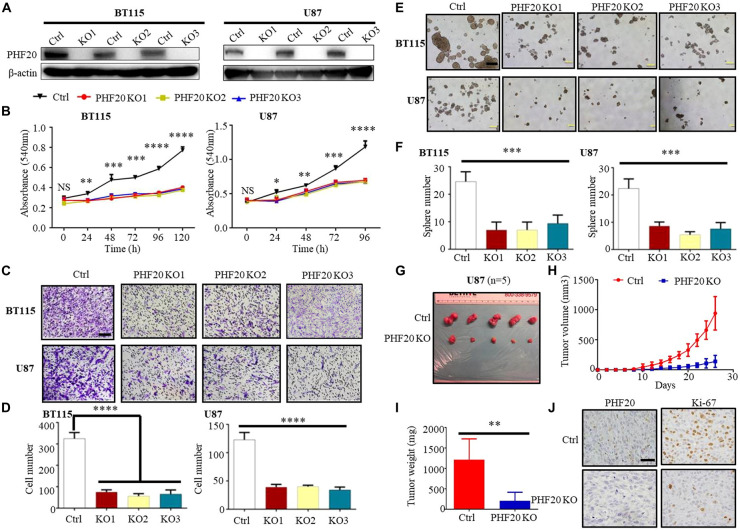
PHF20 is highly expressed in GBM, and increases cellular viability, proliferation and invasiveness of GBM cells *in vitro* and *in vivo*. **(A)** Demonstration of ablation of PHF20 in BT115 and U87 GBM *PHF20* KO cells by western blotting analysis. *PHF20* KO clones were generated with *PHF20* sgRNA #1, #2, and #3. Cells transfected with non-specific sgRNA were used as control. **(B)** A total of 5,000 wild-type (WT) and *PHF20* KO BT115 cells and 10,000 WT and *PHF20* KO U87 cells were plated in a 96-well plate using 200 μl medium. Cell viability was assayed using an MTT assay (540 nm). Both *PHF20* KO BT115 and U87 cells showed significantly reduced cell viability compared to control. **(C)** *PHF20* KO and its control cells were subjected to transwell Matrigel invasion assays. Scale bar, 50 μm. **(D)** The quantification of migrated cells through Matrigel for each cell line. Scale bar, 100 μm. **(E)** A tumor sphere formation assay was performed to assess the self-renewal capacity of WT and *PHF20* KO cells. Five random wells were photographed. **(F)** The quantification of the sphere number after 7 days for each cell line. **(G)** Representative xenografts excised from PHF20 KO and control groups of NSG mice (*n* = 5). **(H)** Growth of tumors following the subcutaneous injection of *PHF20* KO and control cells. **(I)** The tumor weight of subcutaneous xenografts formed by U87 WT and *PHF20* KO cells. The knockout group is remarkably decreased the tumor volume and weight. **(J)** IHC staining of PHF20 and Ki-67 of xenografts. Scale bar, 50 μm. Data are plotted as the mean ± SD of three independent experiments. *P < 0.05; **P < 0.01; ***P < 0.001 compared to the controls using Student’s *t-*test.

To explore the role of PHF20 in GBM tumorigenesis, we firstly determined the PHF20 expression in GBM, the protein level of PHF20 in ten primary GBM cell lines was tested by western blotting: BT115, BT135, BT136, BT139, BT141, BT145, BT147, BT149, BT150, and BT156 (Supplementary Figure 1A). Higher levels of PHF20 expression were found in most GBM cell lines and positive control cells, although little or weak expression in the negative control cells. To determine PHF20 expression in glioma, we firstly measured the expression of PHF20 in glioma tissues by IHC staining. WHO grade I-IV glioma samples from patients were resected in Xiangya Hospital and stained by using a commercially available tissue microarray (Supplementary Figure 1B). We showed a marked increase of PHF20 expression in both the cytoplasm and nucleus of the glioma samples compared to the normal brain tissues (Supplementary Figure 1C). We then established *PHF20* KO cell clones of BT115 and U87 (the details are in Materials and Methods). PHF20 KO efficiency was confirmed by western blotting (Figure 1A), and selected for subsequent experiments. Both *PHF20* KO BT115 and U87 cells showed significantly reduced cell viability compared to control cells (Figure 1B). To examine the effects of PHF20 on cell migration and invasion, we performed a transwell assay with and without Matrigel, using *PHF20* KO BT115 and *PHF20* KO U87 cells. We found that the migration and invasion abilities of GBM cells were significantly reduced compared to the control group (Figures 1C,D and Supplementary Figures 2A,B). It has been previously shown that tumor-initiating cells (TICs) exhibit stem cell-like properties. As such, we determined to examine the contribution of PHF20 to cancer stem-like properties. Since neurospheres are the clonal cell clusters of neural stem cells, we used a neurosphere formation assay to demonstrate that *PHF20* KO significantly impaired the clonogenic capacity and sphere size of BT115 and U87 cells (Figures 1E,F). Indeed, the expression of well-known GBM stemness markers including SOX2, SOX9, OCT4, and NANOG changed significantly under modulation of PHF20 expression (Supplementary Figures 2C,D). To substantiate these *in vitro* observations, we investigated whether knockout of *PHF20* could inhibit the tumorigenic features of GBM cells *in vivo*. *PHF20* KO U87 cells and control cells were subcutaneously injected into NSG mice. Tumor volumes were monitored every other day within 26 days. The ablation of *PHF20* remarkably reduced the tumor volume and weight (Figures 1G–I). Furthermore, the xenograft tumors were resected and processed for immunohistochemical staining (IHC) (Figure 1J). Compared to the normal PHF20 expression in control group, IHC staining indicated that PHF20 was completely deleted in the KO group. The significant reduction of Ki-67 expression levels in *PHF20* KO group further confirmed the impaired tumorigenesis capabilities in *PHF20* KO cells (Figure 1J). Collectively, these results suggest that PHF20 promotes the growth, proliferation and invasiveness of GBM cells.

### Determining the Key Factors Regulated by PHF20 in GBM

**FIGURE 2:**
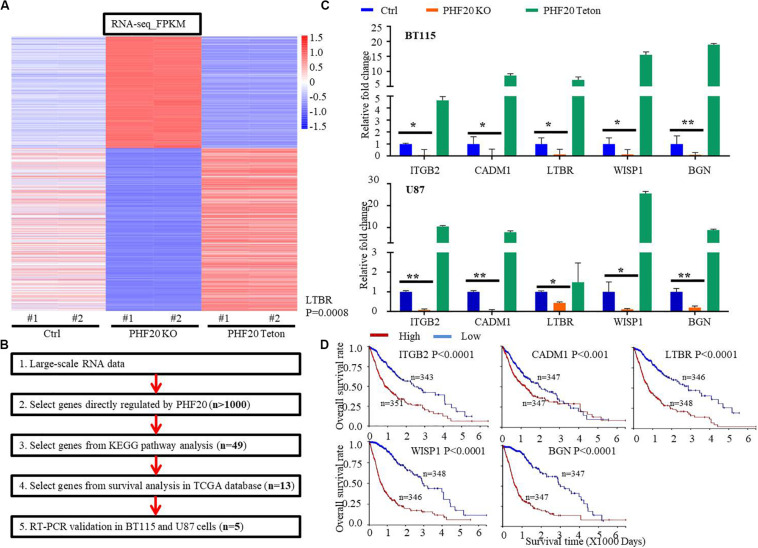
Identification of key downstream factors of PHF20 in GBM. **(A)** RNA-sequencing of *PHF20* KO, *PHF20*-Teton expressing cells and control cells. In control group, cells were transfected with non-specific sgRNA. In PHF20 KO group, cells were transfected with PHF20 sgRNA and PHF20 Teton plasmid (without doxycycline). In PHF20 Teton group, cells were transfected with PHF20 sgRNA and PHF20 Teton plasmid (with doxycycline). **(B)** Flowchart of strategies to explore the key factors of PHF20 according to RNA-sequencing data. **(C)** The expression levels of *ITGB2*, *CADM1*, *LTBR*, *WISP1*, and *BGN* were analyzed by qPCR in PHF20 KO, PHF20 Teton, and control cells. **(D)** The association between *ITGB2*, *CADM1*, *LTBR*, *WISP1*, and *BGN* expression in GBM and the tumor-free survival time of selected patients was analyzed using Kaplan–Meier analysis with the TCGA dataset.

To study the mechanisms by which PHF20 promotes tumor growth and identify the critical downstream factors regulated by PHF20, we performed RNA-sequencing using *PHF20* KO, *PHF20*-Teton, and control cells. To this end, we selected nearly 1,000 genes that were significantly downregulated in *PHF20* KO cells or significantly upregulated in *PHF20* Teton cells, or vice versa (Figure 2A). We then performed Kyoto Encyclopedia of Genes and Genomes (KEGG) pathway analysis for the selected 1,000 genes. Based on the resulting KEGG analysis, 11 KEGG pathways with positive significance were selected, such as Pathways in Cancer, PI3K-Akt signaling pathway, Wnt signaling pathway, and ECM-receptor interaction (Supplementary Table 3). We then selected genes that were directly regulated by PHF20 or genes enriched in the significant KEGG pathways for further functional validation. Through these processes, we narrowed down our targets to 49 genes (Supplementary Figure 2A). Survival analysis in glioma patients was then performed for all 49 genes. We found only 13 of these genes that were significant to the survival of glioma patients (Figure 2B). We further measured the gene expression in *PHF20* KO cells by real-time PCR, and found that only ITGB2, CADM1, LTBR, WISP1, and BGN were consistent in both cell lines (Figure 2C and Supplementary Figure 2B). The high expression of these 5 genes was strongly correlated with a poor median overall survival (OS) in GBM patients based on the TCGA database (*P* < 0.001) (Figure 2D). Taken together, these results suggest that ITGB2, CADM1, LTBR, WISP1, and BGN are the key factors regulated by PHF20 in GBM.

### PHF20 Regulates the Expression of WISP1 by Binding to Its Promoter

**FIGURE 3:**
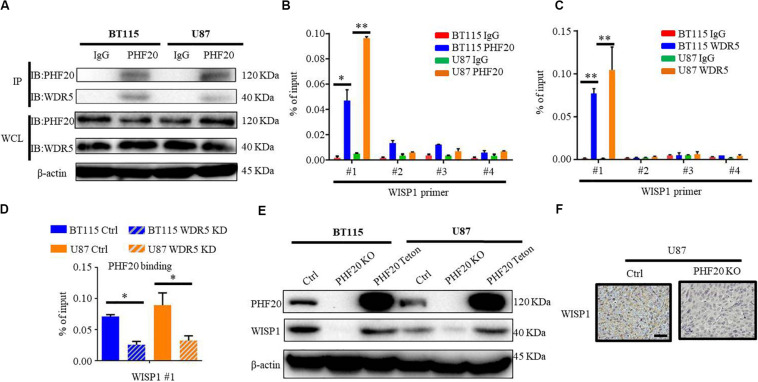
PHF20 regulates the expression of WISP1 by binding to its promoter. **(A)** BT115 and U87 cell extracts were immunoprecipitated with anti-PHF20 antibody, followed by immunoblotting with anti-WDR5 antibody. **(B)** Analysis of PHF20 binding to the promoter regions of *WISP1* in glioma cells by ChIP–qPCR assay with PHF20-specific antibody. The data are presented as fold enrichment relative to input DNA. ChIP-qPCR primers are listed in Supplementary Table 2. **(C)** Analysis of WDR5 binding to the promoter regions of *WISP1* in glioma cells by ChIP–qPCR assay with WDR5-specific antibody. The data are presented as fold enrichment relative to input DNA. **(D)** ChIP-qPCR analysis of WDR5 binding to the promoter region of *WISP1* in WDR5 knockdown cells. The rate of PHF20 binding to the WISP1 promoters decreased in WDR5 KD BT115 and U87 cells compared to the control cells. **(E)** Western blot analysis of PHF20 and WISP1 expression in *PHF20* KO, *PHF20* Teton, and relative control cells. **(F)** IHC staining of WISP1 in xenografts. Scale bar, 50 μm.

To further elucidate how PHF20 regulates the expression of downstream genes, we hypothesized that PHF20 regulates downstream genes by interacting with other epigenetic factors. Our group has demonstrated that PHF20 interacts with WDR5 in induced pluripotent stem cell (iPSCs) (10). However, whether PHF20 and WDR5 interact in GBM remains unclear. To test this possibility, we performed PHF20 and IgG immunoprecipitation of the cell lysates of BT115 and U87 cells, followed by immunoblotting with anti-WDR5 antibody. The western blot analysis showed the interaction of PHF20 with WDR5 (Figure 3A), but they cannot regulate the expression of each other (Supplementary Figures 3A,B). We then designed chromatin immunoprecipitation–quantitative PCR (ChIP–qPCR) assays using both BT115 and U87 cells. PHF20 antibody was used to pull down the chromatin complex with IgG as the negative control. Four pairs of primers against the promoter regions of ITGB2, CADM1, LTBR, WISP1, and BGN were used. The ChIP–qPCR experiments revealed the strong combine of PHF20 on *WISP1* promoters (Figure 3B). However, the bind of PHF20 was not detected at the other five gene promoter regions (Supplementary Figure 3B). Consistently, we generated the same data using WDR5 to pull down the chromatin complex (Figure 3C and Supplementary Figure 3B). Furthermore, the rate of PHF20 binding to the *WISP1* promoters decreased in *WDR5* KD BT115 and U87 cells, compared to the control cells (Figure 3D). The western blotting showed that PHF20 KO could significantly decrease WISP1 expression, while overexpressing PHF20 remarkably upregulated WISP1 expression (Figure 3E). IHC staining of the xenografts revealed that WISP1 was reduced in the PHF20 KO group compared to the control group (Figure 3F), suggesting that PHF20 directly regulates WISP1 expression by interacting with WDR5.

### PHF20 Indirectly Regulates BGN Expression Through WISP1

**FIGURE 4:**
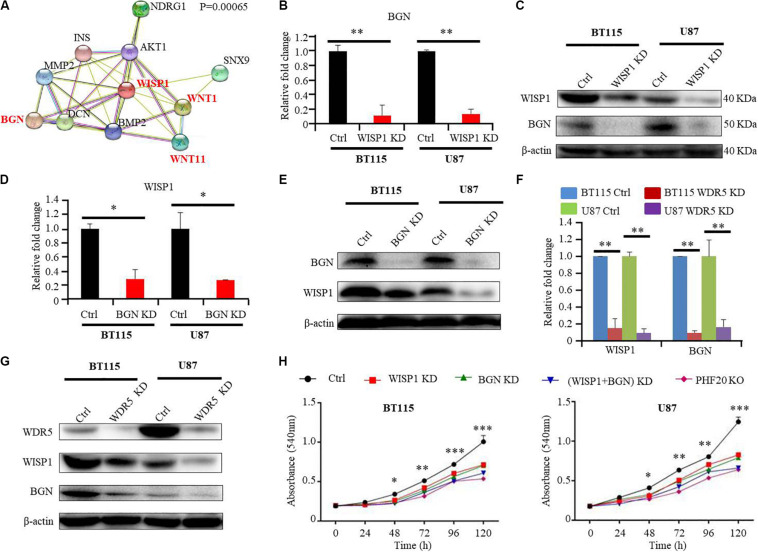
PHF20 indirectly regulates BGN expression through WISP1. **(A)** Protein interaction analysis (PPI) of WISP1. **(B)** The expression level of *BGN* was analyzed by qPCR in *WISP1* KD and control glioma cells. Cells transfected with *WISP1* shRNA or control shRNA were regarded as *WISP1* KD cell or control cell, respectively. **(C)** Western blot analysis of *BGN* in *WISP1* KD and control glioma cells. **(D)** The expression level of *WISP1* was analyzed by qPCR in *BGN* KD and control glioma cells. Cells transfected with *BGN* shRNA or control shRNA were regarded as *BGN* KD cell or control cell, respectively. **(E)** Western blot analysis of *WISP1* in *BGN* KD and control glioma cells. **(F)** The expression levels of *WISP1* and *BGN* were analyzed by qPCR in *WDR5* KD and control glioma cells. Cells transfected with *WDR5* shRNA or control shRNA were regarded as *WDR5* KD cell or control cell, respectively. **(G)** Western blot analysis of *WISP1* and *BGN* in *WDR5* KD and control glioma cells. **(H)** Control GBM cells (control shRNA), *WISP1* KD (*WISP1* shRNA), *BGN* KD (*BGN* shRNA), *WISP1* + *BGN* KD (*WISP1* shRNA + *BGN* shRNA), and *PHF20* KO cells (*PHF20* sgRNA) were plated in a 96-well plate using 200 μl medium. Cell viability was assayed using MTT assay (540 nm). All the groups in BT115 and U87 cells showed significantly reduced cell viability compared to the control cells. Scale bar, 50 μm. Data are plotted as mean ± SD of three independent experiments. *P < 0.05; **P < 0.01; ***P < 0.001 compared with controls using Student’s *t-*test.

Since PHF20 only bound to the *WISP1* promoters, we hypothesized that PHF20 may regulate the expression of other genes through WISP1. To test our hypothesis, we subjected WISP1 to Protein Interaction Analysis (PPI). Interestingly, we found that WISP1 was closely related to BGN, a gene downstream of PHF20. Interestingly, both WISP1 and BGN were found to be tightly involved in the Wnt/-Catenin pathway (Figure 4A). Notably, previous studies show that both BGN and WISP-1 are extracellular matrix proteins and interact each other (24, 25). To explore whether BGN and WISP1 act in concert in GBM through their positive feedback loop, we determined the expression correlation between BGN and WISP1 by qPCR and Western-blotting analyses, and found both the mRNA and protein levels of *BGN* decreased in *WISP1* KD glioma cells (Figures 4B,C). Similarity, both the mRNA level and protein level of *WISP1* decreased in *BGN* KD glioma cells (Figures 4D,E). The knockdown of WDR5 decreased both WISP1 and BGN expression (Figures 4F,G), indicating that WISP1 and BGN may form a positive feedback loop and are regulated by PHF20 and WDR5. Both *WISP1* KD, *BGN* KD, (*WISP1* + *BGN*) KD, and *PHF20* KO in BT115 and U87 cells showed significantly reduced cell viability, compared to the control cells (Figure 4H). Importantly, the WISP1 and BGN double knockdown could achieve the same effects as those caused by PHF20 KO, suggesting that WISP1 and BGN play a dominant role in PHF20-induced aggressiveness in GBM cells.

### PHF20 Stabilizes β-Catenin in a WISP1/BGN-Dependent Manner

**FIGURE 5:**
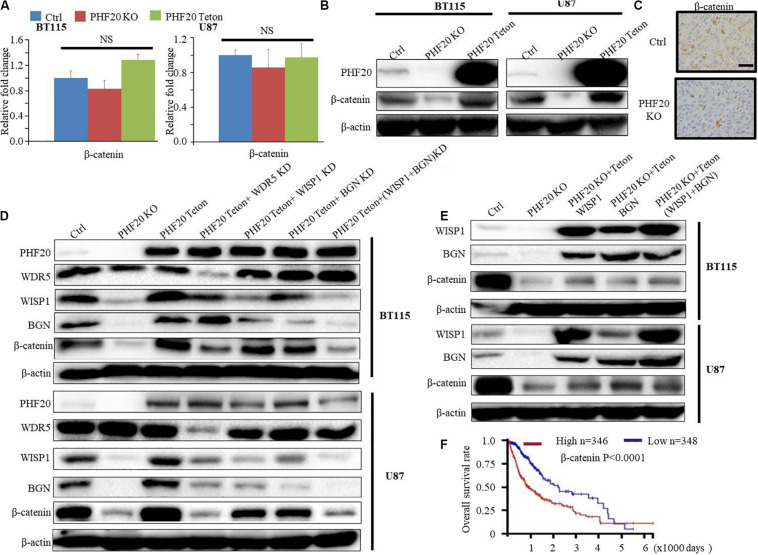
PHF20 stabilizes β-Catenin in a WISP1/BGN-dependent manner. **(A)** The expression level of β*-Catenin* was analyzed by qPCR in *PHF20* KO and control glioma cells. **(B)** Western blot analysis of β*-Catenin* in *PHF20* KO and control glioma cells. **(C)** IHC staining of β-Catenin in xenografts. Scale bar, 50 μm. Control cells (non-specific sgRNA), KO cells (*PHF20* sgRNA). **(D)** Western blot analysis of *PHF20*, *WDR5*, *WISP1*, *BGN*, and β*-Catenin* in *PHF20* KO (*PHF20* sgRNA + *PHF20* Teton, without doxycycline), *PHF20* Teton (*PHF20* sgRNA + *PHF20* Teton + control shRNA, with doxycycline), *PHF20* Teton + *WDR5* KD (*PHF20* sgRNA + *PHF20* Teton + *WDR5* shRNA, with doxycycline), *PHF20* Teton + *WISP1* KD (*PHF20* sgRNA + *PHF20* Teton + *WISP1* shRNA, with doxycycline), *PHF20* Teton + *BGN* KD (*PHF20* sgRNA + *PHF20* Teton + *BGN* shRNA, with doxycycline), *PHF20* Teton + (*WISP1* + *BGN)* KD (*PHF20* sgRNA + *PHF20* Teton + *WISP1* shRNA + *BGN* shRNA, with doxycycline), and control GBM cells (non-specific sgRNA). **(E)** Western blot analysis of *WISP1*, *BGN*, and β*-Catenin in PHF20* KO (*PHF20* sgRNA + *PHF20* Teton, without doxycycline), *PHF20* KO + *WISP1* Teton (*PHF20* sgRNA + *WISP1* Teton, with doxycycline), *PHF20* KO + *BGN* Teton (*PHF20* sgRNA + *BGN* Teton, with doxycycline), *PHF20* KO + (*WISP1* + *BGN)* Teton (*PHF20* sgRNA + *WISP1* Teton + *BGN* Teton, with doxycycline), and control GBM cells (non-specific sgRNA). **(F)** The association between β*-Catenin* expression in GBM and the tumor-free survival time of selected patients was analyzed using Kaplan–Meier analysis with the TCGA dataset.

Because the PPI analysis of WISP1 showed that both WISP1 and BGN are involved in the Wnt/Wnt/β-Catenin pathways, we investigated the relationship between PHF20 and β-Catenin. Our RNA-sequencing data did not show β-Catenin as the downstream gene of PHF20. We further validated our result using RT-PCR and found there was very little change after PHF20 KO or PHF20 Teton compared to the control cells (Figure 5A). However, the results of the western blot analysis of β-Catenin expression in *PHF20* KO, *PHF20* Teton, and control cells showed that β-Catenin was significantly reduced in the *PHF20* KO group but increased in the *PHF20* Teton group (Figure 5B). Similarly, the IHC staining of β-Catenin in the xenografts was much weaker in the *PHF20* KO group than that in the control group (Figure 5C), indicating that PHF20 upregulates the protein level of β-Catenin. To determine whether this occurred in a WISP1- and (or) BGN-dependent manner, we tested WDR5 KD alone, WISP1 KD alone, BGN KD alone, and double WISP1 and BGN KD in *PHF20* Teton GBM cells. We found that even after successfully overexpressing PHF20, the expression of β-Catenin could not be rescued after WISP1 and (or) BGN knockdown (Figure 5D). Consistently, we ectopically induced the expression of *WISP1* and *BGN* alone or together in *PHF20* KO cells. We then confirmed the expression by western blot analysis (Figure 5E) and found that the expression of β-Catenin could be partially rescued when WISP1 and BGN alone or together were ectopically expressed in *PHF20* KO cells. These findings indicate that PHF20 may stabilize β-Catenin protein at least partially in a WISP1/BGN-dependent manner. Furthermore, we also checked the survival analysis of β-Catenin and found the high expression is correlated with poor prognosis in patients with glioma (Figure 5F). Therefore, our study has identified PHF20 as a key factor of GBM growth and development via regulation of β-Catenin through a WISP1 and BGN dependent mechanism, thus serving as a crucial biomarker for GBM diagnosis and a therapeutic target for treatment.

## Discussion

Current strategies used for the treatment of GBM include surgery, radiotherapy, chemotherapy, immunotherapy, and direct treatment of tumors. Despite intensive conventional post-surgery therapies, due to the aggressive nature of this cancer, the prognosis of GBM patients remains poor. An incomplete study of GBM dedifferentiation has decelerated the development of novel therapeutic strategies for GBM. The elucidation of novel key factors that regulate the stem cell-like phenotype of GBM cells is of great significance to increase our knowledge of this type of cancer, for the individually precise treatment of this disease.

Plant homeodomain finger-containing protein 20 was confirmed to be an autoantibody in GBM patients (13). Interestingly, glioma patients with PHF20 autoantibody have significantly better prognosis than patients without this autoantibody, indicating that a potential therapeutic option for the treatment of GBM is to develop immunotherapy or targeting therapy against PHF20. Our previous study revealed that PHF20 plays an important role in somatic reprogramming through epigenetic regulation. The down-regulation or knockdown of PHF20 expression in somatic cells was found to markedly inhibit the activation of the endogenous Oct4 gene, thus decreasing the efficiency of the iPSCs (10). Further, PHF20 has been found to be highly expressed in lung cancer, malignant adenoma, brain glioma, and other tumor tissues, and is closely related to the development and progression of tumors (8, 11, 12). Similarity, a previous study performed a bioinformatics analysis to determine the function of PHF20 in various cancers using the TCGA database and found that PHF20 was highly expressed in colon cancer, cervical cancer, bladder cancer, and lung cancer, a result that was further experimentally verified in lung cancer cells (15). In addition, it has been documented that PHF20 is involved in tumorigenesis by inhibiting p53 expression (9). Moreover, our recent study showed that PHF20 collaborates with PARP1 and activates two critical downstream factors, SOX2 and OCT4, subsequently promoting the growth and invasion of NB cells (16). This study identified a novel function of PHF20 in its promoting the essential characters of malignant GBM. Despite some limitation in the different time points of our transwell assay and lack of more dilution in ELISA assay, our study discovered the phenotype that PHF20 plays a vital role in the proliferation, migration and sphere formation activities in GBM cells. Mechanistically, PHF20 interacts with the WD repeat domain 5 (WDR5) and directly binds to the promoter regions of WISP1. Subsequently, WISP1 and BGN act as functional partner to regulate the degradation of β-Catenin, which plays a vital role in GBM progression. As a co-transcription factor, PHF20 combines with other transcription factors. WDR5 is a well-known chaperone of PHF20 and has been reported to function as an oncogene in many cancers, including glioma (26–33). Our previous study demonstrated that PHF20 interacts with WDR5 and plays an important role in cellular reprogramming and neuroblastoma aggressiveness. WISP1 had been identified as an oncogene in GBM. Glioma stem cells endogenously secreted WISP1 within glioma tumor microenvironment, and the secretion of WISP1 promoted the glioma development through a glioma-associated macrophages dependent mechanism (34). Knockdown of WISP1 or inhibition WISP1/β-Catenin markedly inhibited the malignant characters of GBM (34, 35). Intriguingly, several studies indicated that WISP1 was functionally related to BGN in tumor development (24, 25, 36). BGN is also one of the downstream genes of PHF20. On the basis of these findings, we suggest the functional interplay of WISP1-BGN in GBM development, and further demonstrate that the expression and function of WISP1-BGN are regulated by PHF20-WDR5 axis.

To the best of our knowledge, this study is the first to show the novel function of the PHF20-WDR5 axis in GBM. To elucidate the critical downstream genes of PHF20 in GBM, we performed RNA sequencing using two independent *PHF20* KO, *PHF20* Teton, and control GBM cells. As a result, 5 key downstream genes were identified: ITGB2, CADM1, LTBR, WISP1, and BGN. Notably, it was found that PHF20 and WDR5 directly bind to the WISP1 transcription sequence at the same site, but not the other four genes. By carrying out an in-depth investigation of the functions of WISP1 and BGN, we found a large number of reports linking these genes with the Wnt/β-Catenin signaling pathway (25, 37–42). Consistently, our PPI analysis of WISP1 indicated that both WISP1 and BGN are closely associated with the Wnt/β-Catenin signaling pathway. As a classic oncogene, the function of β-Catenin has been fully verified in GBM. Indeed, β-Catenin was strongly correlated with a poor median overall survival in GBM patients on the TCGA database.

**FIGURE 6:**
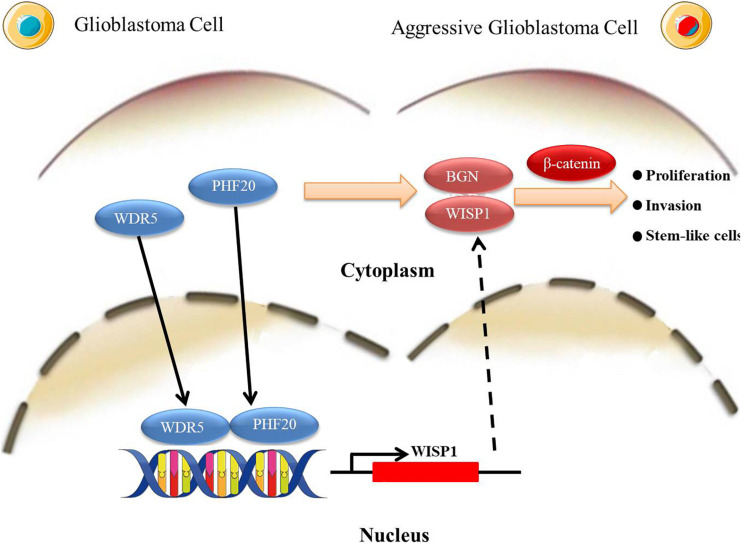
A schematic illustration of a working model. *PHF20* interacts with *WDR5* for the activation of *WISP1* directly and *BGN* indirectly, which in turn stabilizes β*-Catenin* and confers stem cell-like traits to glioma cells. The activation of these key factors by *PHF20* may lead to rapid cell proliferation and aggressive stem cell-like phenotypes in GBM.

Based on our findings in this study, we propose a working model to illustrate the interplay of PHF20 and WDR5 modulates the WISP1 promoters, leading to *WISP1* activation, which in turn results in the subsequent induction of *BGN*. This increases and stabilizes the β-Catenin protein levels and promotes GBM malignant transformation (Figure 6). Accordingly, the stabilization of β-Catenin will coordinate with WNT1 to promote the transcriptional activation of WISP1 in nucleus (43), which forms a multi-interactive feedback loop. Similarly, the contribution of PHF20 on the stabilization of other proteins had also been reported to function as an effector protein of p53 double lysine methylation that eliminated ubiquitination and stabilizes p53 (44). The detailed molecular mechanism of PHF20 and WISP1/BGN on the stabilization of β-Catenin protein warrants further investigation. Based on these results, our findings provide new opportunities to identify new therapeutic approaches via the pharmacological inhibition of PHF20 activity or the targeting of PHF20 for immunotherapy in GBM.

## Conclusion

The expression of PHF20 is elevated in glioma samples and associated with potential poor prognosis in patients with glioma. Ablation of PHF20 dramatically impairs the malignancies of GBM cell lines. PHF20 and WDR5 cooperate to regulate β-Catenin via the mediation of WISP1 and BGN promotes GBM malignant transformation.

## Data Availability Statement

The original contributions presented in the study are publicly available. This data can be found here: https://www.ncbi.nlm.nih.gov/bioproject/PRJNA660891/.

## Ethics Statement

The animal study was reviewed and approved by Institutional Animal Care and Use Committee (Houston Methodist Research Institute).

## Author Contributions

QM and WL performed the experiments and wrote the manuscript. CJ and JS performed the bioinformatics analysis. CX revised the manuscript. QL and RW designed the experiments, interpreted the data, wrote the manuscript, and provided supervision. All authors contributed to the article and approved the submitted version.

## Conflict of Interest

The authors declare that the research was conducted in the absence of any commercial or financial relationships that could be construed as a potential conflict of interest.

## Footnotes

1
https://rdrr.io/cran/pheatmap/

## Supplementary Material

Supplementary Figure 1 **(A)** Western blot analysis of PHF20 in ten primary GBM cell lines. Negative control cells were normal glial cell line HEB cells, and induced pluripotent stem cells (iPS cells) were used as positive control cells. **(B)** IHC staining of PHF20 in glioma of WHO grade I–IV from patients and comparison with normal brain tissues resected in Xiangya Hospital. Original magnification, ×100. Scale bar, 50 μm. **(C)** The upper part is the representative intensity at each level of PHF20 IHC staining. Lower part is the intensity of IHC staining of PHF20 in cytoplasm and nuclei of glioma tissue array. Scale bar, 20 μm. ^∗^*P* < 0.05; ^∗∗^*P* < 0.01; ^∗∗∗^*P* < 0.001.

Supplementary Figure 2 **(A)** *PHF20* KO and its control cells were subjected to transwell migration assays. **(B)** The quantification of migrated cells for each cell line. **(C)** The expression levels of stemness markers including *SOX2*, *SOX9*, *OCT4*, and *NANOG* were analyzed by qPCR in *PHF20* KO, *PHF20* Teton, and relative control cells. **(D)** Western blot analysis of *SOX2*, *SOX9*, *OCT4*, and *NANOG* expression in *PHF20* KO, *PHF20* Teton, and relative control cells.

Supplementary Figure 3 **(A)** List of 49 target genes in step 3 related to Figure 3B. **(B)** qPCR validation in PHF20 KO, PHF20 Teton, and relative control cells.

Supplementary Figure 4 **(A,B)** Western blot analysis of *WDR5* in *PHF20* KO, *PHF20* Teton, and relative control cells. And western blot analysis of *PHF20* in *WDR5* KD and relative control cells. PHF20 and *WDR5* cannot regulate the expression of each other. **(C)** PHF20 and WDR5 were not detected in ITGB2, LTBR, CADM1 and BGN promoter sites.

Supplementary Table 1 Real-time PCR Primers.

Supplementary Table 2 ChiP-PCR Primers.

Supplementary Table 3 List of significant KEGG pathway in Figure 2.

## Abbreviations

BGN: biglycan
CSCs: cancer stem cells
GBM: glioblastoma
GSCs: glioblastoma stem cells
KD: knock-down
KO: knockout
PHF20: plant homeodomain finger-containing protein 20
sgRNA: single-guide RNA
WDR5: WD repeat-containing protein 5
WISP1: WNT1 inducible signaling pathway protein 1.
