# Corrigendum: PHF20 promotes glioblastoma cell malignancies through a *WISP1/BGN*-dependent pathway

**DOI:** 10.3389/fonc.2023.1157694

**Published:** 2023-03-22

**Authors:** Qianquan Ma, Wenyong Long, Changsheng Xing, Chongming Jiang, Jun Su, Helen Y. Wang, Qing Liu, Rongfu Wang

**Affiliations:** ^1^ Department of Neurosurgery in Xiangya Hospital, Central South University, Changsha, China; ^2^ Department of Neurosurgery in the Third Hospital of Peking University, Peking University, Beijing, China; ^3^ Center for Inflammation and Epigenetics, Houston Methodist Research Institute, Houston, TX, United States; ^4^ Department of Medicine, Keck School of Medicine, University of Southern California, Los Angeles, CA, United States; ^5^ Department of Pediatrics, Children’s Hospital of Los Angeles, Keck School of Medicine, University of Southern California, Los Angeles, CA, United States; ^6^ Norris Comprehensive Cancer Center, Keck School of Medicine, University of Southern California, Los Angeles, CA, United States

**Keywords:** PHF20, glioblastoma, WISP1, BGN, cancer stem cell-like traits, epigenetic regulation

In the original article, there was an error in **Figure 1** as published. In **Figure 1C**, the images labeled “PHF20 KO2” and “PHF20 KO3” in the row for “BT115” show an overlapping field of view. The corrected version of **Figure 1** and its caption appears below.

**Figure 1:**
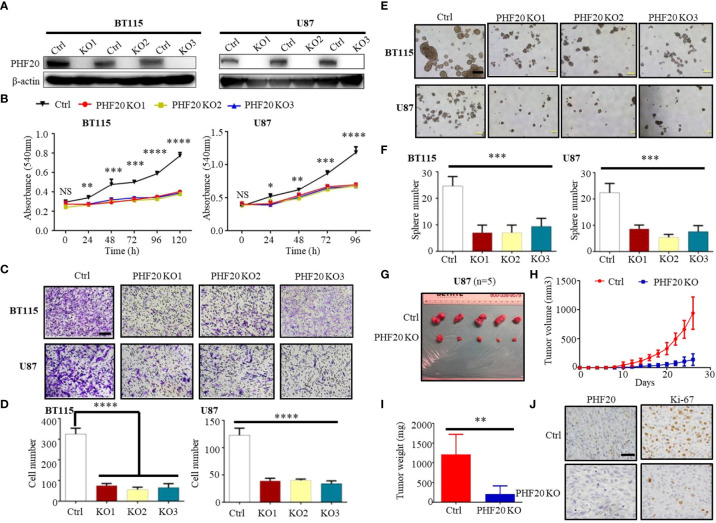
PHF20 is highly expressed in GBM, and increases cellular viability, proliferation and invasiveness of GBM cells *in vitro* and *in vivo*. **(A)** Demonstration of ablation of PHF20 in BT115 and U87 GBM PHF20 KO cells by western blotting analysis. PHF20 KO clones were generated with PHF20 sgRNA #1, #2, and #3. Cells transfected with non-specific sgRNA were used as control. **(B)** A total of 5,000 wild-type (WT) and PHF20 KO BT115 cells and 10,000 WT and PHF20 KO U87 cells were plated in a 96-well plate using 200 μl medium. Cell viability was assayed using an MTT assay (540 nm). Both PHF20 KO BT115 and U87 cells showed significantly reduced cell viability compared to control. **(C)** PHF20 KO and its control cells were subjected to transwell Matrigel invasion assays. Scale bar, 50 μm. **(D)** The quantification of migrated cells through Matrigel for each cell line. Scale bar, 100 μm. **(E)** A tumor sphere formation assay was performed to assess the self-renewal capacity of WT and PHF20 KO cells. Five random wells were photographed. **(F)** The quantification of the sphere number after 7 days for each cell line. **(G)** Representative xenografts excised from PHF20 KO and control groups of NSG mice (n = 5). **(H)** Growth of tumors following the subcutaneous injection of PHF20 KO and control cells. **(I)** The tumor weight of subcutaneous xenografts formed by U87 WT and PHF20 KO cells. The knockout group is remarkably decreased the tumor volume and weight. **(J)** IHC staining of PHF20 and Ki-67 of xenografts. Scale bar, 50 μm. Data are plotted as the mean ± SD of three independent experiments. *P < 0.05; **P < 0.01; ***P < 0.001 compared to the controls using Student’s t-test.

The authors apologize for these errors and state that they do not change the scientific conclusions of the article in any way. The original article has been updated.

