# Supplementary figures and images for: PHF20 Promotes Glioblastoma Cell Malignancies Through a WISP1/BGN-Dependent Pathway
Source: Front Oncol. 2020 Oct 6;10:573318. doi: 10.3389/fonc.2020.573318 (PMC7574681; doi:10.3389/fonc.2020.573318)

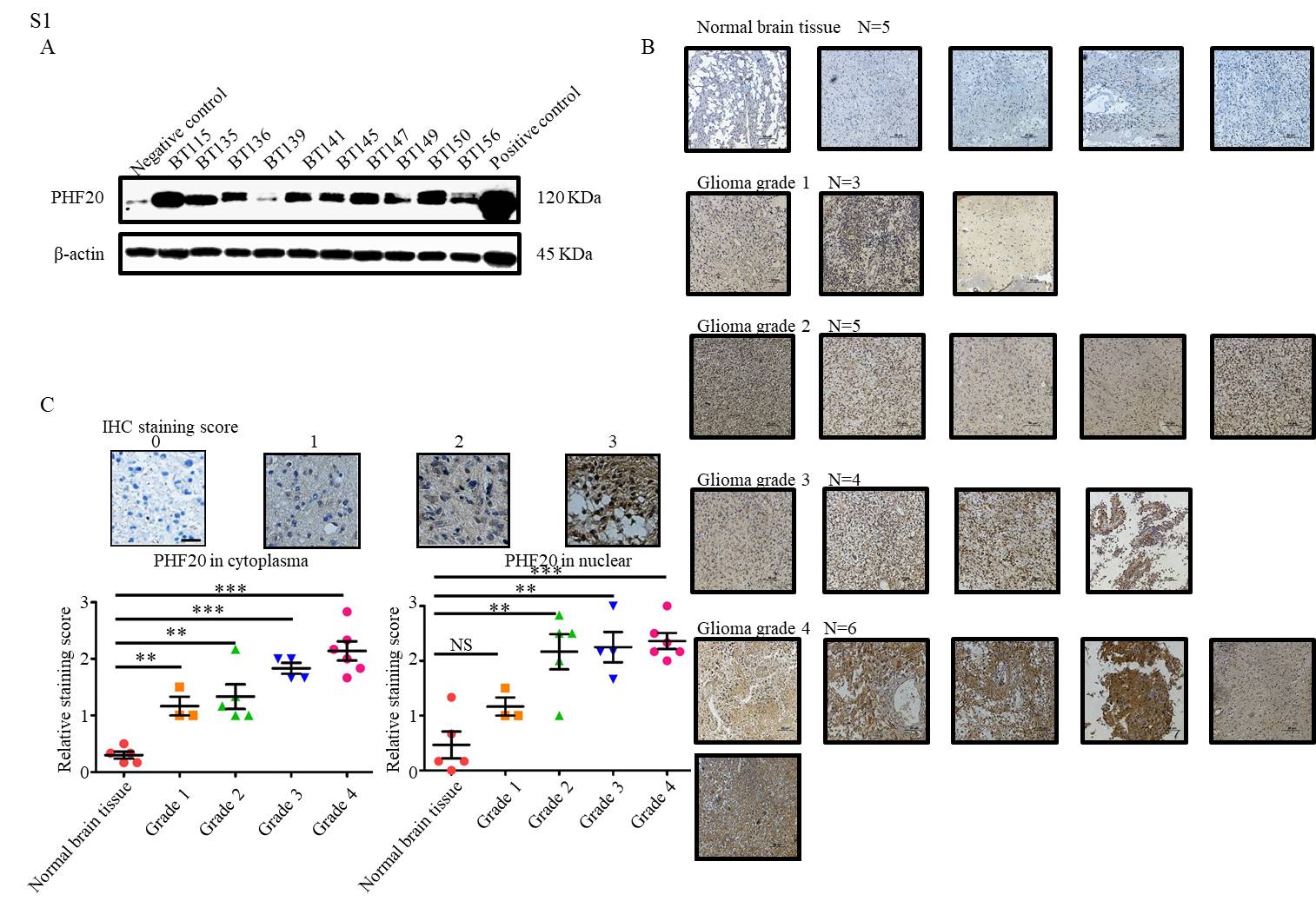

Supplement: Supplementary Figure 1 — (A) Western blot analysis of PHF20 in ten primary GBM cell lines. Negative control cells were normal glial cell line HEB cells, and induced pluripotent stem cells (iPS cells) were used as positive control cells. (B) IHC staining of PHF20 in glioma of WHO grade I–IV from patients and comparison with normal brain tissues resected in Xiangya Hospital. Original magnification, ×100. Scale bar, 50 μm. (C) The upper part is the representative intensity at each level of PHF20 IHC staining. Lower part is the intensity of IHC staining of PHF20 in cytoplasm and nuclei of glioma tissue array. Scale bar, 20 μm. ∗P < 0.05; ∗∗P < 0.01; ∗∗∗P < 0.001. [file Image_1.JPEG]

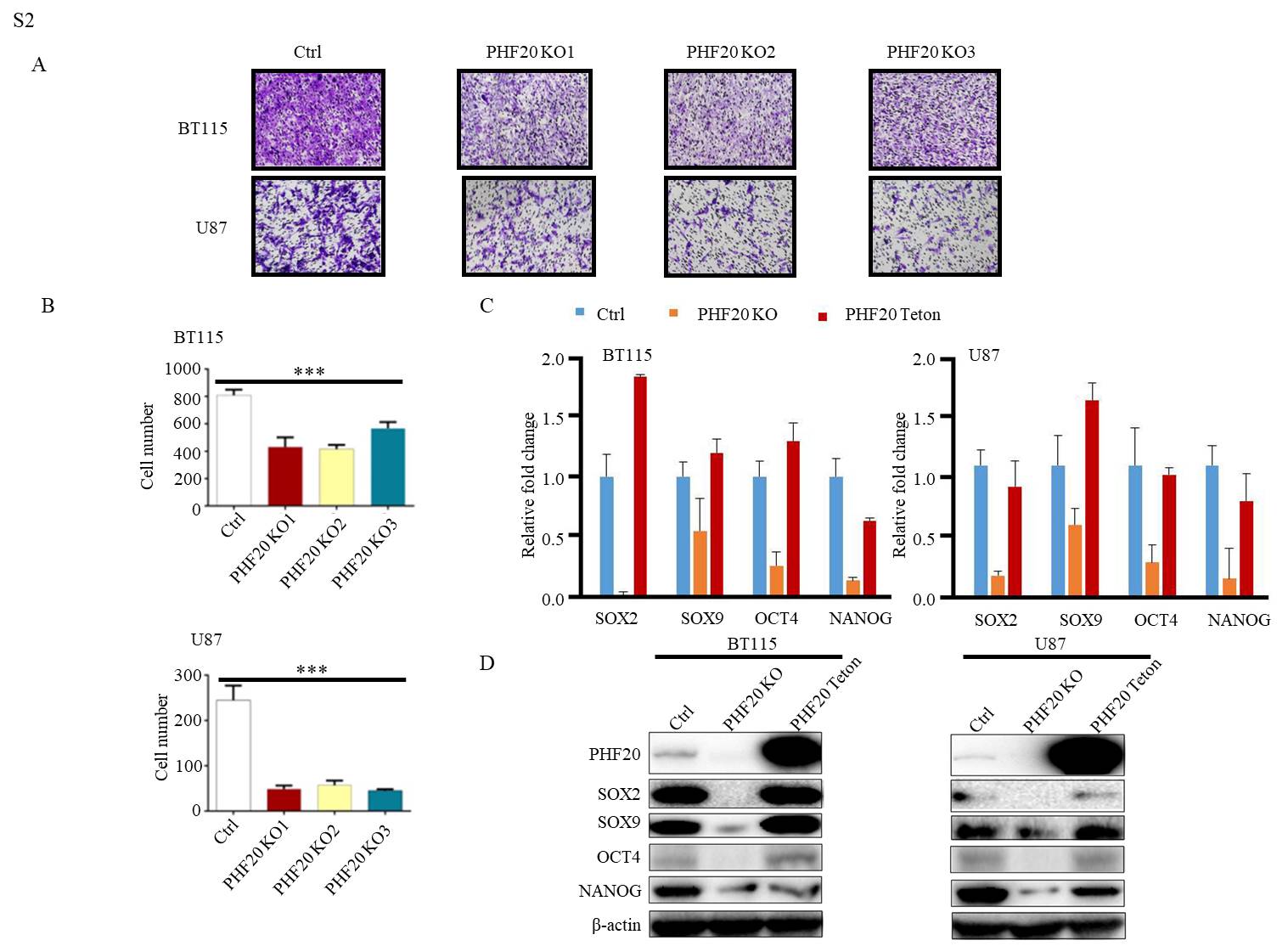

Supplement: Supplementary Figure 2 — (A) PHF20 KO and its control cells were subjected to transwell migration assays. (B) The quantification of migrated cells for each cell line. (C) The expression levels of stemness markers including SOX2, SOX9, OCT4, and NANOG were analyzed by qPCR in PHF20 KO, PHF20 Teton, and relative control cells. (D) Western blot analysis of SOX2, SOX9, OCT4, and NANOG expression in PHF20 KO, PHF20 Teton, and relative control cells. [file Image_2.JPEG]

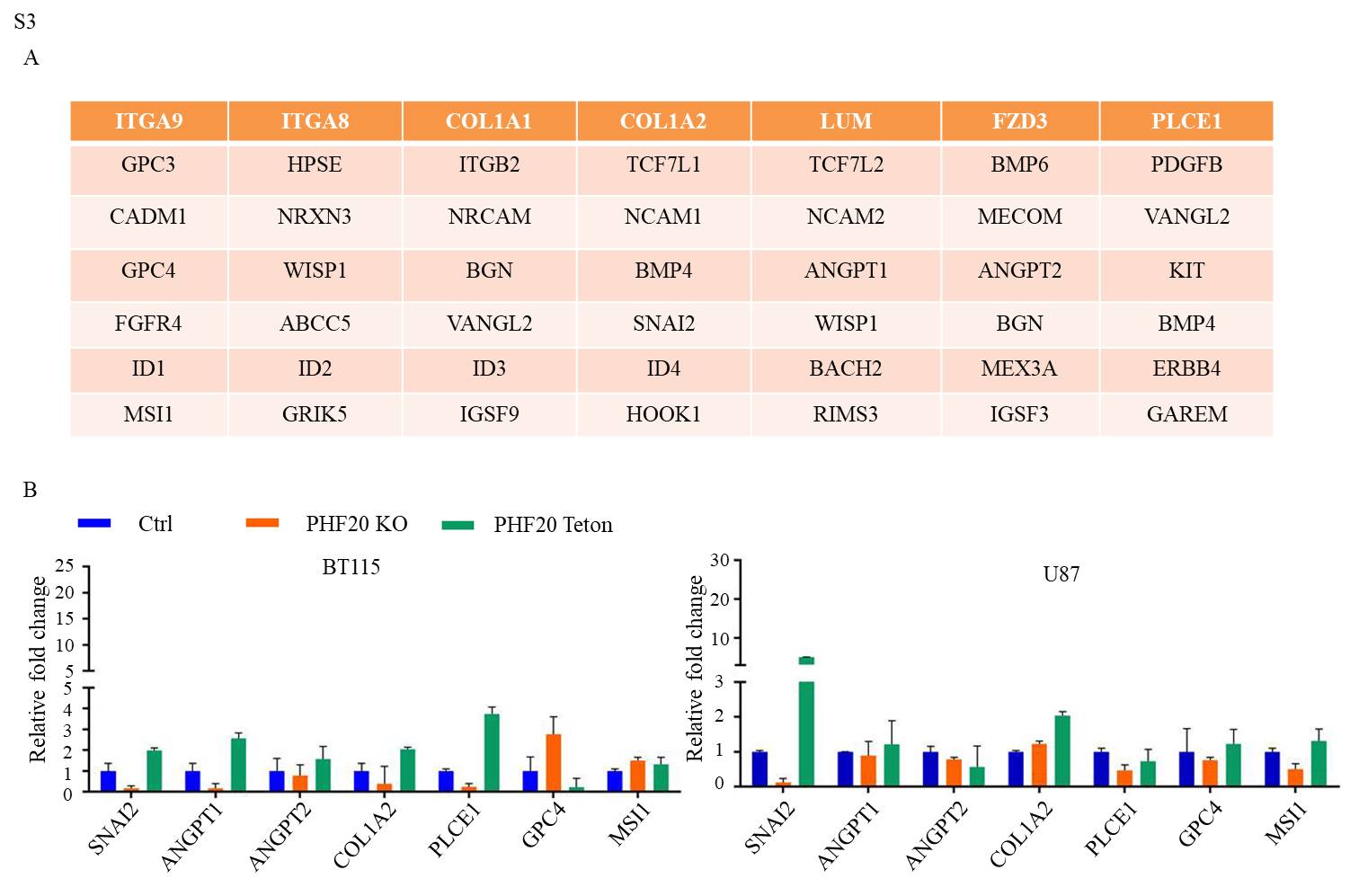

Supplement: Supplementary Figure 3 — (A) List of 49 target genes in step 3 related to Figure 3B. (B) qPCR validation in PHF20 KO, PHF20 Teton, and relative control cells. [file Image_3.JPEG]

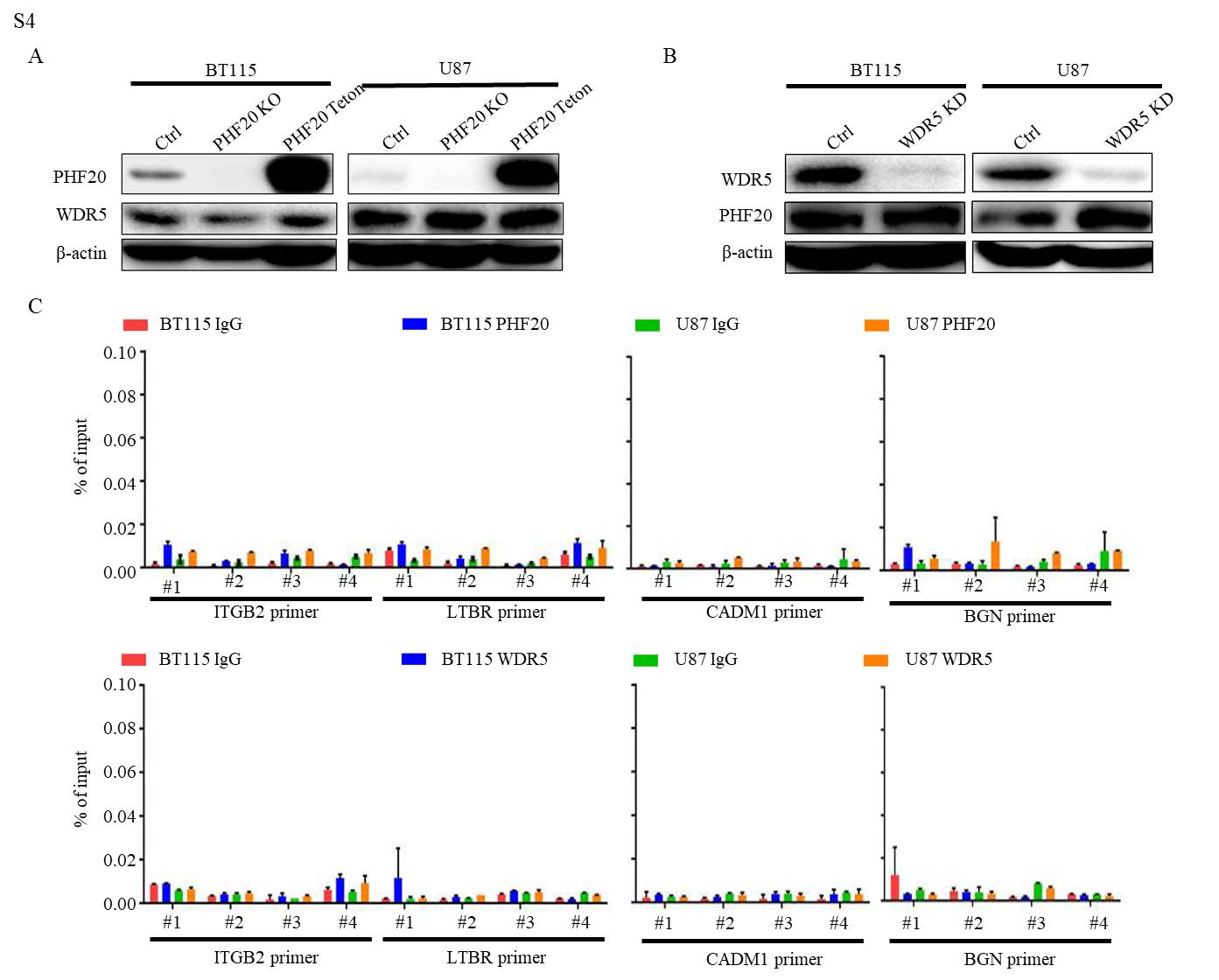

Supplement: Supplementary Figure 4 — (A,B) Western blot analysis of WDR5 in PHF20 KO, PHF20 Teton, and relative control cells. And western blot analysis of PHF20 in WDR5 KD and relative control cells. PHF20 and WDR5 cannot regulate the expression of each other. (C) PHF20 and WDR5 were not detected in ITGB2, LTBR, CADM1 and BGN promoter sites. [file Image_4.JPEG]
