# Supplementary material for: PHF20 Promotes Glioblastoma Cell Malignancies Through a WISP1/BGN-Dependent Pathway
Source: Front Oncol. 2020 Oct 6;10:573318. doi: 10.3389/fonc.2020.573318 (PMC7574681; doi:10.3389/fonc.2020.573318)
Supplement: Supplementary Table 1 — Real-time PCR Primers. [file Data_Sheet_1.docx]

**Table 1**

**Real-time PCR Primers**

| WISP1-F | AGACCCACTGAAATGACC |
| --- | --- |
| WISP1-R | AACCTCCATCTTCCTACC |
| BGN-F | GAGACCCTGAATGAACTCCACC |
| BGN-R | CTCCCGTTCTCGATCATCCTG |
| CADM1-F | ATGGCGAGTGTAGTGCTGC |
| CADM1-R | GATCACTGTCACGTCTTTCGT |
| ITGB2-F | TGCGTCCTCTCTCAGGAGTG |
| ITGB2-R | GGTCCATGATGTCGTCAGCC |
| LTBR-F | CACAAGCAAACGGAAGACCC |
| LTBR-R | GACGCAGTGGTTGTTACCCT |
| GPC4-F | GTGGGAAATGTGAACCTGGAA |
| GPC4-R | CGAGGGACATCTCCGAAGG |
| MSI1-F | GGGACTCAGTTGGCAGACTAC |
| MSI1-R | CTGGTCCATGAAAGTGACGAA |
| PLCE1-F | GCTTCTTAACACGGGACTTGG |
| PLCE1-R | CTTCAAGGGCATTGTGCTCTC |
| COL1A2-F | GTTGCTGCTTGCAGTAACCTT |
| COL1A2-R | AGGGCCAAGTCCAACTCCTT |
| ANGPT2-F | AACTTTCGGAAGAGCATGGAC |
| ANGPT2-R | CGAGTCATCGTATTCGAGCGG |
| SNAI2-F | CGAACTGGACACACATACAGTG |
| SNAI2-R | CTGAGGATCTCTGGTTGTGGT |
| ANGPT1-F | AGCGCCGAAGTCCAGAAAAC |
| ANGPT1-R | TACTCTCACGACAGTTGCCAT |
| β-Catenin-F | CCCAGCGTCGTCTGCTTTA |
| β-Catenin-R | CGATTCGCTCTCCCCGTAAC |

**Table 2**

**ChiP-PCR Primers**

| ITGB2-CHIP-1F | TCCAATGTCCTCCATGTCCG |
| --- | --- |
| ITGB2-CHIP-1R | GGGGACAGTGTGTGGGAGAA |
| ITGB2-CHIP-2F | TCGCAGCTCACTTTTACGGA |
| ITGB2-CHIP-2R | AGGGGATGACCAATGTGCAG |
| ITGB2-CHIP-3F | CAGATACCACCCACAGGACAC |
| ITGB2-CHIP-3R | GTGGGGAGCTGTCCCTTCT |
| ITGB2-CHIP-4F | GTGTTCAGAGACAAGGCGGG |
| ITGB2-CHIP-4R | TTCCATGGGGGCTTCCTAAG |
| CADM1-CHIP-1F | TGAGCATACCCTCCTCGATCT |
| CADM1-CHIP-1R | GGTTCTGAGATTTTCCAGTCGG |
| CADM1-CHIP-2F | AAAGCACGCATGCACTTCTC |
| CADM1-CHIP-2R | TGCGGGAGTGGAGAGTAAGA |
| CADM1-CHIP-3F | TCTCCAGTCGTCGGTCTGAT |
| CADM1-CHIP-3R | GGCGGGTCTAGCTTCTTGTA |
| CADM1-CHIP-4F | CTGTGATTGGTCTGCCCGGA |
| CADM1-CHIP-4R | GCCCGAGCGGACAGCTAA |
| LTBR-CHIP-1F | CAGCTAAATGTAGCCGCATCC |
| LTBR-CHIP-1R | CAGGTAGTTCCAGTGCTCGT |
| LTBR-CHIP-2F | CACCGCTGCCCAGGAC |
| LTBR-CHIP-2R | CATGGCGGCCACTCGG |
| LTBR-CHIP-3F | TGGGTCACGGGGGCTC |
| LTBR-CHIP-3R | CCAGTTTTCCTGGGGGACAG |
| LTBR-CHIP-4F | CAGCTAAATGTAGCCGCATCC |
| LTBR-CHIP-4R | CAGGTAGTTCCAGTGCTCGT |
| WISP1-CHIP-1F | ATGAGCAGAGACGGCAAACA |
| WISP1-CHIP-1R | AGGCTCAGGTTACAGCATCAC |
| WISP1-CHIP-2F | GGTGGACTGAAAGCCACCTC |
| WISP1-CHIP-2R | TGGTTTTCATTTGCCCTCTTGC |
| WISP1-CHIP-3F | GCAGCCTGAAGAAATGACAGG |
| WISP1-CHIP-3R | GATACTGTTACCCCAGGCAGG |
| WISP1-CHIP-4F | AAAGACCTAGGCAGGATGACC |
| WISP1-CHIP-4R | CAAGGCTATGACTTCACACTAGG |
| BGN-CHIP-1F | TCTCACTCCATCTAAACTCTTACCC |
| BGN-CHIP-1R | TAGTGTCCCCTTCTTCCCTCA |
| BGN-CHIP-2F | GCTCTAGCCACTGGAGGAAC |
| BGN-CHIP-2R | GGGTTACCCCACCAAGACTC |
| BGN-CHIP-3F | CCGTAAGTGACCAGCACAGG |
| BGN-CHIP-3R | GTCCCCTCGGACATGAGAAC |
| BGN-CHIP-4F | CCACCTCCTTGCTTAGGCCC |
| BGN-CHIP-4R | GCTGCTAGGCCCATGAGTG |

**Table 3**

**KEGG pathway with statistical significance.**

| Term of pathway | Count of genes | P value | genes |
| --- | --- | --- | --- |
| hsa04512:ECM-receptor interaction | 15 | 7.65E-05 | COL4A1, COL3A1, HSPG2, COL5A2, COL5A1, ITGA9, ITGAV, ITGA8, COL6A3, COL1A2, COL1A1, LAMB1, COL11A1, FN1, THBS4 |
| hsa05205:Proteoglycans in cancer | 23 | 3.08E-04 | PRKCA, FZD8, ERBB4, ERBB3, LUM, HSPG2, TLR4, FZD3, DCN, MMP2, PPP1CB, ITPR1, PLAUR, IGF1R, PLCE1, CDKN1A, GPC3, PLCG1, HPSE, ITGAV, PTCH1, AKT3, FN1 |
| hsa04390:Hippo signaling pathway | 19 | 4.10E-04 | BMP4, FZD8, GDF6, SOX2, WWC1, ITGB2, FZD3, SNAI2, PPP1CB, TCF7L2, TCF7L1, LLGL2, CTNNA2, ID2, CTGF, ID1, AMOT, AXIN2, BMP6 |
| hsa04510:Focal adhesion | 23 | 4.67E-04 | PRKCA, COL4A1, PDGFB, PGF, TLN2, COL3A1, PPP1CB, COL5A2, COL5A1, ITGA9, IGF1R, RAC3, ITGAV, JUN, ITGA8, COL6A3, COL1A2, COL1A1, LAMB1, COL11A1, AKT3, FN1, THBS4 |
| hsa04514:Cell adhesion molecules (CAMs) | 18 | 5.62E-04 | F11R, PTPRF, CADM1, NRXN3, SELL, ITGB2, LRRC4C, ALCAM, NRCAM, NCAM1, ITGA9, NCAM2, ITGAV, ITGA8, CNTN1, CLDN2, VCAN, HLA-DRA |
| hsa04520:Adherens junction | 12 | 6.25E-04 | MAP3K7, PTPRJ, IGF1R, PTPRF, SORBS1, RAC3, PTPN1, SNAI2, SNAI1, TCF7L2, TCF7L1, CTNNA2 |
| hsa04066:HIF-1 signaling pathway | 13 | 0.00295 | PRKCA, IGF1R, CDKN1A, LTBR, CDKN1B, PLCG1, EDN1, EGLN3, HK2, TLR4, ANGPT1, ANGPT2, AKT3 |
| hsa05200:Pathways in cancer | 33 | 0.00311 | PDGFB, PGF, EGLN3, KIT, MMP2, TCF7L2, MMP1, TCF7L1, IGF1R, RAC3, ITGAV, TGFA, AXIN2, LAMB1, AKT3, FN1, PRKCA, BMP4, FZD8, COL4A1, SKP2, FZD3, MECOM, CTNNA2, DAPK1, CDKN1A, CDKN1B, PLCG1, LPAR6, JUN, PTCH1, GNAS, GNB4 |
| hsa04151:PI3K-Akt signaling pathway | 29 | 0.00588 | PRKCA, FGFR4, COL4A1, PDGFB, PGF, CSF1, COL3A1, TLR4, KIT, COL5A2, COL5A1, ITGA9, IGF1R, CDKN1A, CDKN1B, LPAR6, ITGAV, ITGA8, COL6A3, COL1A2, GNB4, ANGPT1, COL1A1, LAMB1, COL11A1, ANGPT2, AKT3, FN1, THBS4 |
| hsa04310:Wnt signaling pathway | 16 | 0.00772 | PRKCA, FZD8, VANGL2, FZD3, TCF7L2, TCF7L1, MAP3K7, WISP1, CTNNBIP1, GPC4, DKK1, RAC3, JUN, PRICKLE2, LRP6, AXIN2 |
| hsa04550:Signaling pathways regulating pluripotency of stem cells | 13 | 0.04245 | BMP4, IGF1R, FZD8, FGFR4, SMAD9, ID2, ID1, SOX2, ID4, FZD3, ID3, AXIN2, AKT3 |
